# Written patient information materials used in general practices fail to meet acceptable quality standards

**DOI:** 10.1186/s12875-020-1085-6

**Published:** 2020-02-01

**Authors:** Nicole Posch, Karl Horvath, Kerstin Wratschko, Jasper Plath, Richard Brodnig, Andrea Siebenhofer

**Affiliations:** 1 Institute of General Practice and Evidence-Based Health Services Research, Medical University of Graz, Auenbruggerplatz 20/3, Graz, Austria; 2 Clinical Department of Endocrinology and Metabolism, Medical University of Graz, Auenbruggerplatz 15, Graz, Austria; 3 Institute of General Practice, Goethe University Frankfurt am Main, Theodor-Stern-Kai 7, Frankfurt, Germany

**Keywords:** Patient information materials, Health literacy, General practice, EQIP

## Abstract

**Background:**

Patient information materials and decision aids are essential tools for helping patients make informed decisions and share in decision-making. The aim of this study was to investigate the quality of the written patient information materials available at general practices in Styria, Austria.

**Methods:**

We asked general practitioners to send in all patient information materials available in their practices and to answer a short questionnaire. We evaluated the materials using the Ensuring Quality Information for Patients (EQIP-36) instrument.

**Results:**

A total of 387 different patient information materials were available for quality assessment. These materials achieved an average score of 39 out of 100. The score was below 50 for 78% of all materials. There was a significant lack of information on the evidence base of recommendations. Only 9 % of the materials provided full disclosure of their evidence sources. We also found that, despite the poor quality of the materials, 89% of general practitioners regularly make active use of them during consultations with patients.

**Conclusion:**

Based on international standards, the quality of patient information materials available at general practices in Styria is poor. The vast majority of the materials are not suitable as a basis for informed decisions by patients. However, most Styrian general practitioners use written patient information materials on a regular basis in their daily clinical practice. Thus, these materials not only fail to help raise the health literacy of the general population, but may actually undermine efforts to enable patients to make shared informed decisions. To increase health literacy, it is necessary to make high quality, evidence-based and easy-to-understand information material available to patients and the public. For this, it may be necessary to set up a centralized and independent clearinghouse.

## Background

The health literacy of 36% of the U.S. population is either basic or below basic [1]. The situation in Europe is similar, with one study finding that the knowledge of 48% of Europeans is inadequate or problematical. This is particularly true of Austria where the health knowledge of 56% of the population is inadequate or problematical [2]. Patient information materials and decision aids are an important means of raising the health literacy of patients [3]. They also provide the basis for informed participatory decision-making in questions relating to health and medical therapies. A recently updated Cochrane-Review [4] has shown that decision aids raise knowledge about alternative treatments, give patients the feeling that they are well informed and provide them with a clearer idea of the benefits and risks of a medical intervention. Decision aids enable patients to be more involved in the decision-making process [4].

Criteria defining high quality patient information materials are well established and instruments to analyze structural quality have long been available [5–9]. Participatory decision making and an adequate level of health information are not only in the interest of patients [3, 10] but also a legal requirement in many countries, including Austria [11].

General practitioners are not only the first port of call of patients [12], they are also one of the main sources of health information [13]. The quality of patient information materials and decision aids that are available at the doctor’s practice are therefore of particular importance.

Existing studies of the quality of patient information materials generally only deal with specific diseases [14–19] and rarely take into account how the information is used in general practices [15, 19].

The systematic collection of data and the evaluation of the structural quality of the patient information materials and decision aids that are available to patients in primary care have not previously been carried out in Austria.

The principal aim of this study was:

- To determine the quality of the patient information materials used in Austrian general practices.

Further aspects that were investigated were:

- To what extent are patient information materials used on a day-to-day basis in general practices?
- Who publishes such patient information materials and are differences in quality dependent on the publisher?
- Are evidence sources provided and cited in patient information materials?

## Methods

The study was carried out with the help of the Styrian general practitioners (GPs) that had indicated in a previous survey that they would be interested in participating in general practice research [20]. In a short questionnaire, the doctors were asked to provide basic demographic information and details on their use of patient information materials (see Additional file 1: questionnaire). They were then asked to return the questionnaire, along with one copy of each of the patient information materials and decision aids that are available in their practice.

Information materials were considered to be relevant to this study when “declarations were made on the consequences of diseases, the effects of preventive and diagnostic measures, and on therapies for diseases.” Information materials were not taken into account when they referred to the provision of services at specific sites or were purely for advertising purposes.

The long version of the Ensuring Quality Information for Patients (EQIP-36) instrument was used to evaluate the remaining patient information materials [21]. The instrument includes three dimensions: a total of 18 criteria describe the content of patient information materials (e.g. description of a medical problem, treatment alternatives, and information on the benefits and risks of a therapy), six criteria concern data identification (e.g. references to financing, sources and publisher) and twelve criteria deal with the structure of the materials (e.g. legibility, use of graphics, whether readers are addressed respectfully). A score on a scale of 0 to 100 is used to determine the quality of health information, with a score of 0 indicating that no criterion was satisfied and a score of 100 showing that all criteria were completely fulfilled [21].

First of all, relevant metadata was extracted (title, publisher, date of publication, and volume of the information materials). The EQIP instrument was then used to analyze the materials and a score was calculated.

One reviewer evaluated each of the information materials, while a second independent reviewer controlled a randomly selected sample of 10 % of the materials. Significant differences in the evaluation were resolved via discussion.

Microsoft Excel 2016 MSO (Version 1712) was used to perform a descriptive statistical analysis of the data pseudoanonymously.

## Results

**Fig. 1:**
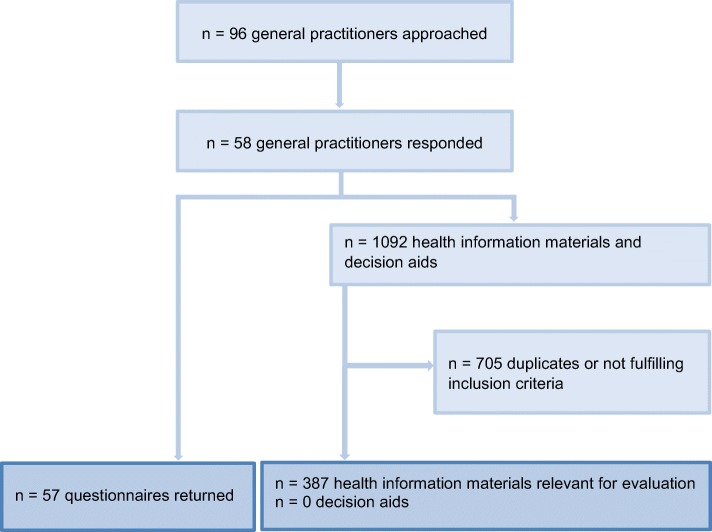
Information procurement

Of the 96 GPs we contacted, 57 completed the questionnaires and 58 returned a copy of each of the patient information materials they used in their practices (response rate 60%). Overall, 1092 leaflets and brochures were sent back. After excluding identical materials and information materials that did not fulfil the inclusion criteria, 387 written patient information materials remained for evaluation (Fig. 1). The GPs sent back between 0 and 90 such materials. The average number returned was 19.

The demographic characteristics of the participating GPs can be found in Additional file 2: Table S1.

### Use of information materials

A total of 55 GPs (96%) said they used the submitted materials on a day-to-day basis, and 51 (89%) made active use of them during consultations. In 53 (93%) of the practices, the doctors only provided the patients with selected information materials, whereas in four practices, the doctors made all the materials at their disposal available. Reasons provided for not making patient information materials available to patients were content (68%), too little space in the general practice (58%), publisher (32%) and lack of integrity or intelligibility in the opinion of the doctor (19%).

### Characteristics of the information materials

Of a total of 387 leaflets and brochures, 268 provided general information on diseases, 216 information on medications and medicinal products, 72 information on tests, operations screenings and interventions, 34 information on services, five information on follow-up treatments, and 170 on other topics. Some of the materials dealt with several subjects. None of the information materials fulfilled the criteria required to be considered a decision aid.

Overall, 372 (96%) of the brochures and leaflets provided information on the publishers. Most of the materials (60%) were published by producers of pharmaceuticals and other medical products. Other publishers were professional societies, associations, and initiatives (14%), health and social security funds (6%), media enterprises and publishing groups (3%) and others (11%). A further 10 % of the materials had been written by the doctors themselves.

### EQIP evaluation of patient information materials

#### EQIP score

**Fig. 2:**
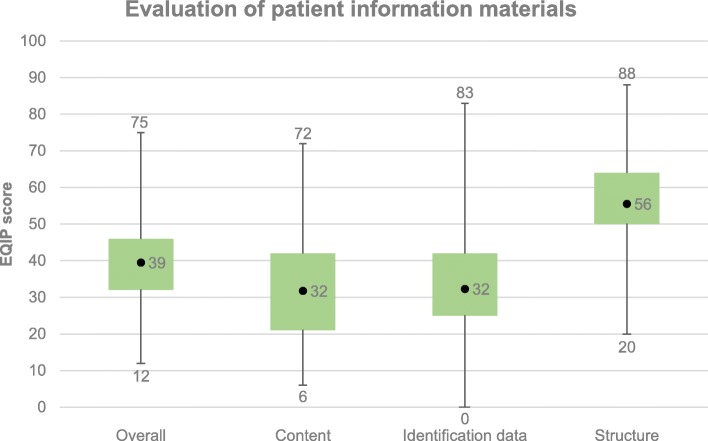
Boxplot-Whisker of the average, minimum and maximum values for the information materials, overall and broken down into individual dimensions

Figure 2 shows that the information materials had an average overall EQIP score of 39, with 303 (78%) of them scoring below 50. The highest score was 75. Average scores were also calculated for the individual dimensions and are displayed in Fig. 2. The average scores on the dimensions “content” and “identification” were both 32, while the average score was 56 on the dimension “structure”. Poor scores on the dimension “content” generally reflected a lack of information on benefits, risks and side effects. Low scores on the dimension “identification” were mostly due to a lack of information on the date of issue and any updates, a lack of information on financing, and few details on whether patients were involved in the preparation of the brochure or leaflet. The source of evidence was rarely mentioned at all. Higher scores were achieved on the dimension “structure”, which reflected the use of simple language, a comprehensible structure, appropriate graphics and a clear layout.

**Fig. 3:**
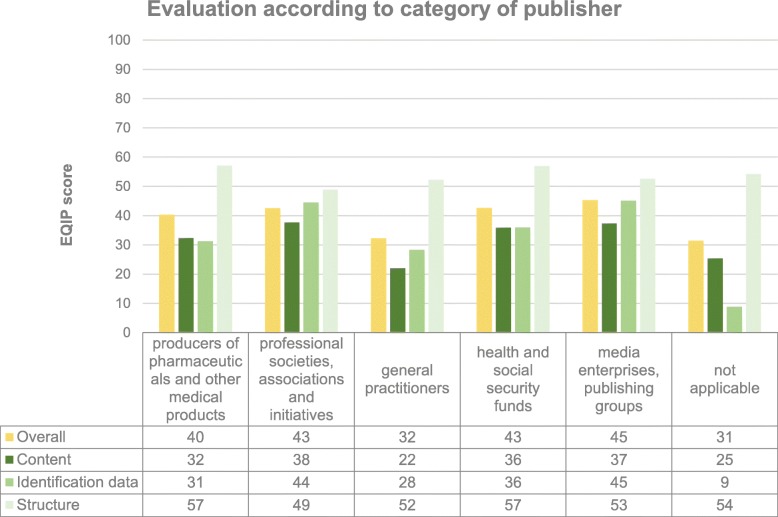
Averages for all information materials, overall and broken down into individual dimensions for the most common categories of publisher

The overall average score for all categories of publisher was below 50. A separate evaluation of the materials was also carried out for individual publisher categories (see Fig. 3), whereby the average was 40–45 for media enterprises and publishing groups, health and social security funds, professional societies, associations and initiatives and producers of pharmaceuticals and other medical products. The overall average score was significantly lower for brochures written by the doctors themselves (32) and brochures that gave no indication of the publisher (31). Fig. 3 provides a breakdown of the individual dimensions (content, identification and structure) for each category of publisher.

#### Disclosure of sources of evidence

No sources of evidence were disclosed for 327 (84%) of the leaflets and brochures. Some source information was disclosed for 26 (7%) of the materials (for individual citations, illustrations, text passages) and only 34 (9%) provided full disclosure of sources. The frequency of source disclosure varied insignificantly between categories of publisher.

## Discussion

Patient information materials are used in physician-patient communication in Styrian family practices on a daily basis. Most of these materials are provided by producers of pharmaceuticals and medical products. However, about a tenth of the materials are written by the doctors themselves. Overall the materials are of low quality. They especially lack information on the potential benefits and harms of specified medical services, on financing, and on the evidence base for the provided medical information and recommendations.

Our results are similar to those identifed in several international studies, which also identified serious quality shortcomings in patient information materials [13, 16, 18, 19, 22]. However, these studies investigated information materials on specific diseases, while we considered all the information materials available to patients in general practices, regardless of topic. The results of the present study thus strengthen and complement what has previously been described.

Patients increasingly wish to play an active role in the decision-making process [22, 23], and written health information can serve as a helpful supplement in physician-patient consultations [3]. However, many currently available information materials do not provide satisfactory and sufficiently balanced information, and it is often impossible for both patients and physicians to assess the reliability of provided recommendations. While high quality written health information materials can, in principle, increase patients´ knowledge, improve their experience of healthcare and strengthen their participation in the decision-making process [13], many of the currently available information materials do not. Thus, shared decision making, based on the best available evidence and comprehensible information, is still hindered by the poor quality of information materials.

Providers of health information should prepare and update health information materials on the basis of existing quality criteria. In particular, benefits, risks and side effects should be provided, and references should be included. In order to ensure the quality of health information is high, minimum standards for reliable health information should further be established by law.

It would be helpful if GPs and doctors in training were trained to evaluate patient information materials themselves. However, in view of the large number of available materials, it is unlikely that doctors will be able to find the time to select the best quality materials themselves. It is therefore necessary to establish a central, independent clearinghouse to examine and prepare brochures and leaflets based on international criteria for “good patient information” [9].

A Cochrane Review has shown that a combination of verbal and written information can improve patients´ knowledge and satisfaction more effectively than verbal information alone [24]. Doctors should therefore be taught to use health information materials as a supportive tool in medical consultations during their medical training.

Since May 2018, the implementation of these suggestions has been progressing as part of a new project financed by the Styrian health fund (“EVI-project: Evidence-based health information to increase health literacy”) [25].

It is necessary that the broader public be made aware that the use of information materials of inferior quality can lead to a number of problems.

The question remains as to what extent the objective quality criteria used by the EQIP instrument are the same as patients’ subjective expectations of information materials, and this is a limitation of our study.

Furthermore, most of the GPs that participated in our study had already shown an interest in general practice research, and it is possible that the involvement of another sample of GPs would have led to different results. However, existing studies on the subject have identified very few differences between GPs that are interested in research and those that are not [26–28].

In their consultations, doctors also use patient information materials they have prepared themselves, or they provide materials as summaries of, for example, diet recommendations and gymnastic exercises. As these materials are generally not designed to be comprehensive, it is unclear whether the EQIP instrument is suitable for evaluating them, especially when specific details on their planned use are not taken into consideration.

## Conclusions

Patient information materials are essential tools for supporting patient-physician communication, and raising health knowledge, and they provide a valuable basis for shared decision making. To achieve this, however, it is necessary that they are reliable and of good quality. As this is not true of the majority of current information materials, it is important to find ways to ensure that high quality materials are available in sufficient quantities in the future.

## Supplementary information

**Additional file 1:** Questionnaire.

**Additional file 2: Table S1.** Demographic characteristics of the participating GPs.

## Data Availability

The datasets used and/or analyzed for the current study are available from the corresponding author on reasonable request.

## Abbreviations

EQIP: Ensuring Quality Information for Patients
GP(s): General practitioner(s)
