# Supplementary material for: Written patient information materials used in general practices fail to meet acceptable quality standards
Source: BMC Fam Pract. 2020 Feb 1;21:23. doi: 10.1186/s12875-020-1085-6 (PMC6995648; doi:10.1186/s12875-020-1085-6)
Supplement: Supplementary file 1 — Additional file 1: Questionnaire. [file 12875_2020_1085_MOESM1_ESM.docx]

We ask you to return the **questionnaire** together with your **collected information materials** after filling out.

**General information to your person and your medical activity**

| Years of professional experience as general practitioner | O less than 5 years |
| --- | --- |
|  | O 5 to15 years |
|  | O 15 to 30 years |
|  | O more than 30 years |
| Sex | O female  O male |
| You work as | O GP with a contract with Austrian Health Insurance  O GP with a contract with obligatory insurance (others than Austrian Health insurance) |
|  | O private GP without a contract |
| Do you proactively sort out any information materials? | O yes  O no |
| If yes, how many do you sort out? | O less than the half  O approximately the half  O more than the half |
| What are your reasons for sorting out information materials? (multiple responses possible) | O insufficient space  O topic  O editor  O _ _ _ _ _ _ _ _ _ _ _ _ |
| Do you use any information materials in the course of your consultations? | O yes  O no |
| Do you actively hand out any information materials to your patients in your practice? | O yes  O no |
| Do you use any information materials in the course of your consultations that you created yourself? | O yes  O no |
| Do you actively hand out any information materials to your patients in your practice that you created yourself? | O yes  O no |

**Thank you for your support!**

Please feel free to leave your **address**/ practice stamp for **simplification** of our analyses, **of course only if you like**:
