# Supplementary material for: Written patient information materials used in general practices fail to meet acceptable quality standards
Source: BMC Fam Pract. 2020 Feb 1;21:23. doi: 10.1186/s12875-020-1085-6 (PMC6995648; doi:10.1186/s12875-020-1085-6)
Supplement: Supplementary file 2 — Additional file 2: Table S1. Demographic characteristics of the participating GPs. [file 12875_2020_1085_MOESM2_ESM.docx]

| **Demographic characteristics** | | **N (%)** |
| --- | --- | --- |
| Sex | female | 29 (51) |
|  | male | 28 (49) |
| Years of professional experience | <5 years  5-15 years  15-30 years  >30 years | 2 (4)  5 (9)  26 (45)  24 (42) |
